# Neighborhood and Individual Socioeconomic Disadvantage and Survival Among Patients With Nonmetastatic Common Cancers

**DOI:** 10.1001/jamanetworkopen.2021.39593

**Published:** 2021-12-17

**Authors:** En Cheng, Pamela R. Soulos, Melinda L. Irwin, Elizabeth M. Cespedes Feliciano, Carolyn J. Presley, Charles S. Fuchs, Jeffrey A. Meyerhardt, Cary P. Gross

**Affiliations:** 1 Department of Chronic Disease Epidemiology, Yale School of Public Health, New Haven, Connecticut; 2 Division of Research, Kaiser Permanente Northern California, Oakland, California; 3 Cancer Outcomes, Public Policy, and Effectiveness Research Center, Yale Cancer Center, New Haven, Connecticut; 4 Yale Cancer Center, Smilow Cancer Hospital, New Haven, Connecticut; 5 Division of Medical Oncology, Department of Internal Medicine, Ohio State University, Columbus; 6 Hematology and Oncology Product Development, Genentech & Roche, South San Francisco, California; 7 Department of Medical Oncology, Dana-Farber Cancer Institute, Boston, Massachusetts

## Abstract

**Importance:**

Disadvantaged neighborhood-level and individual-level socioeconomic status (SES) have each been associated with suboptimal cancer care and inferior outcomes. However, independent or synergistic associations between neighborhood and individual socioeconomic disadvantage have not been fully examined, and prior studies using simplistic neighborhood SES measures may not comprehensively assess multiple aspects of neighborhood SES.

**Objective:**

To investigate the associations of neighborhood SES (using a validated comprehensive composite measure) and individual SES with survival among patients with nonmetastatic common cancers.

**Design, Setting, and Participants:**

This prospective, population-based cohort study was derived from the Surveillance, Epidemiology, and End Results–Medicare database from January 1, 2008, through December 31, 2011, with follow-up ending on December 31, 2017. Participants included older patients (≥65 years) with breast, prostate, lung, or colorectal cancer.

**Exposures:**

Neighborhood SES was measured using the area deprivation index (ADI; quintiles), a validated comprehensive composite measure of neighborhood SES. Individual SES was assessed by Medicare-Medicaid dual eligibility (yes vs no), a reliable indicator for patient-level low income.

**Main Outcomes and Measures:**

The primary outcome was overall mortality, and the secondary outcome was cancer-specific mortality. Hazard ratios (HRs) for the associations of ADI and dual eligibility with overall and cancer-specific mortality were estimated via Cox proportional hazards regression. Statistical analyses were conducted from January 23 to April 15, 2021.

**Results:**

A total of 96 978 patients were analyzed, including 25 968 with breast, 35 150 with prostate, 16 684 with lung, and 19 176 with colorectal cancer. Median age at diagnosis was 76 years (IQR, 71-81 years) for breast cancer, 73 years (IQR, 70-77 years) for prostate cancer, 76 years (IQR, 71-81 years) for lung cancer, and 78 years (IQR, 72-84 years) for colorectal cancer. Among lung and colorectal cancer patients, 8412 (50.4%) and 10 486 (54.7%), respectively, were female. The proportion of non-Hispanic White individuals among breast cancer patients was 83.7% (n = 21 725); prostate cancer, 76.8% (n = 27 001); lung cancer, 83.5% (n = 13 926); and colorectal cancer, 81.1% (n = 15 557). Neighborhood-level and individual-level SES were independently associated with overall mortality, and no interactions were detected. Compared with the most affluent neighborhoods (ADI quintile 1), living in the most disadvantaged neighborhoods (ADI quintile 5) was associated with higher risk of overall mortality (breast: HR, 1.34; 95% CI, 1.26-1.43; prostate: HR, 1.51; 95% CI, 1.42-1.62; lung: HR, 1.21; 95% CI, 1.14-1.28; and colorectal: HR, 1.24; 95% CI, 1.17-1.32). Individual socioeconomic disadvantage (dual eligibility) was associated with higher risk of overall mortality (breast: HR, 1.22; 95% CI, 1.15-1.29; prostate: HR, 1.29; 95% CI, 1.21-1.38; lung: HR, 1.14; 95% CI, 1.09-1.20; and colorectal: HR, 1.23; 95% CI, 1.17-1.29). A similar pattern was observed for cancer-specific mortality.

**Conclusions and Relevance:**

In this cohort study, neighborhood-level deprivation was associated with worse survival among patients with nonmetastatic breast, prostate, lung, and colorectal cancer, even after accounting for individual SES. These findings suggest that, in order to improve cancer outcomes and reduce health disparities, policies for ongoing investments in low-resource neighborhoods and low-income households are needed.

## Key Points

**Question:**

After accounting for individual socioeconomic disadvantage, is living in a deprived neighborhood associated with worse survival after cancer diagnosis?

**Findings:**

In this cohort study of 96 978 older patients with nonmetastatic breast, prostate, lung, and colorectal cancers, neighborhood-level disadvantage was associated with worse survival even after adjusting for patient-level low income.

**Meaning:**

These findings suggest that in order to improve cancer outcomes and reduce health disparities, policies for ongoing investments in low-resource neighborhoods and low-income households are needed.

## Teaser

This cohort study examines the association of neighborhood and individual socioeconomic disadvantage with survival among older patients with nonmetastatic common cancers.

## Introduction

Socioeconomic status (SES) is a complex concept describing the state of income, wealth, education, occupation, and living conditions for both individuals and their neighborhood or social network.^1,2^ Individual SES has consistently been reported to be an important risk factor for worse survival across a range of cancer types.^3,4,5,6,7,8,9,10,11,12,13,14,15,16^ However, investigators often focus on the associations of neighborhood SES with cancer survival in large national cancer databases in which individual-level SES is not accessible^17,18^; thus, individual-level SES is underexamined in these national databases. Owing to systemic inequalities in opportunity, development, and built environment,^19^ disadvantaged neighborhood SES may result in worse cancer outcomes via multiple pathways. For example, in deprived neighborhoods, lack of physicians and health care resources,^20^ inefficacious referral systems,^21^ low trust in health care providers,^22^ poor social support networks,^23^ and barriers to travel for initial and follow-up care^24^ may together negatively impact survival after cancer diagnosis.^25,26,27,28,29,30,31,32^

Despite the understanding that both individual and neighborhood SES influence cancer outcomes, important knowledge gaps exist. First, it is unclear whether neighborhood SES is independently associated with cancer outcomes after accounting for individual-level SES, or whether there is a synergistic association between these 2 factors.^17,18^ To our knowledge, the interaction of individual-level and neighborhood-level SES with cancer survival has not been previously compared. Second, given the strong connection between income and comorbidity, it is important to explore the association between SES and all-cause mortality as well as cancer-specific mortality. Third, prior analyses have tended to use single-domain SES measures (income, education, poverty, etc) or create overly simplistic composite neighborhood SES measures.^25,26,27,28,29,30,31,32^ However, neighborhood-level SES remains underexamined in general given that these approaches may not accurately capture the whole spectrum of factors that contribute to neighborhood SES. In contrast,^33^ the area deprivation index (ADI) consists of 17 measures of education, employment, housing quality, and poverty originally extracted from long-form US Census data, and offers a theoretical advantage.^34^ One study found a higher ADI (neighborhood disadvantage) to be associated with worse survival after cancer diagnosis, but these patients were diagnosed between 1988 and 1999, and investigators did not adjust for cancer stage or treatment in the analysis.^8^ Thus, new studies are urgently needed to apply more recent cancer data with comprehensive adjustment for factors influencing prognosis.

To better assess cancer outcomes in the context of neighborhood and individual socioeconomic disadvantage, we used (1) the ADI,^34,35^ a comprehensive composite measure of neighborhood SES, and (2) Medicare-Medicaid dual eligibility (DE),^36^ a measure of patient-level low income status. Although DE may not reflect the whole spectrum of individual-level SES, it captures one of the most important aspects of individual SES: personal income.^37^ In addition, it has been consistently validated as an informative and practical tool,^38^ especially given that individual-related SES information is frequently unavailable in large national cancer databases.^17,18^ Using the Surveillance, Epidemiology, and End Results (SEER)–Medicare linked database, we investigated the associations of ADI and DE with survival among elderly patients diagnosed with breast, prostate, lung, and colorectal cancer and assessed whether there was an interaction between individual- and neighborhood-level SES. In the United States, older patients (≥65 years) constitute 62.9% of the cancer population,^39^ and these 4 cancers are among the most commonly diagnosed cancers, accounting for 43.1% of cancer-related deaths in 2021.^40^ Our analysis may inform a better understanding of the relationship between neighborhood and individual SES and cancer outcomes and may support policies for ongoing investments in lower-resource neighborhoods and low-income households.

## Methods

### Data Source and Study Population

The SEER-Medicare database reflects the linkage of 2 large population-based sources of data, which provide detailed demographic, clinical, and cause-of-death information about Medicare beneficiaries (aged ≥65 years) with cancer.^41^ The SEER program covers approximately 27.8% of the US population via 18 population-based cancer registries in 13 states.^42^ Medicare is a national health insurance covering individuals aged 65 years or older and those younger than 65 years with special medical needs.^43^ We used SEER-Medicare data to identify patients diagnosed with nonmetastatic breast (*International Classification of Diseases for Oncology, Third Edition* [*ICD-O-3*] code: 26000), prostate (*ICD-O-3* code: 28010), lung (*ICD-O-3* code: 22030), and colorectal cancer (*ICD-O-3* codes: 21041-21052) between January 1, 2008, and December 31, 2011. Rather than using more recent years of diagnosis, we selected this time frame to give sufficient follow-up time, because our sample comprises patients with nonmetastatic diseases who may live for many years after cancer diagnosis. In addition, patients must have been enrolled in Medicare Parts A and B fee-for-service coverage from 2 years prior to diagnosis through 1 year after. This time frame ensured that we would have access to complete claims in order to assess prediagnosis comorbidities and receipt of cancer treatment. Patients must have had at least 1 claim billed to Medicare during this fee-for-service enrollment window. Thus, included patients were age 67 years or older at time of diagnosis. eFigure 1 in the Supplement represents the derivation of the final included populations for each cancer. The Yale Institutional Review Board approved this study as exempt human research because SEER-Medicare data were deidentified, and informed consent from patients was not required. This study followed the Strengthening the Reporting of Observational Studies in Epidemiology (STROBE) reporting guideline.

### Area Deprivation Index and Medicare-Medicaid Dual Eligibility

We used the latest version of the ADI available at the time of this study (2018) to measure patients’ neighborhood SES via linked ZIP codes.^44^ The ADI ranges from 1 to 100 and is presented in national percentile rankings, with higher scores indicating higher levels of neighborhood socioeconomic disadvantage. In line with previous studies,^33,45,46,47,48^ we categorized the ADI measure into quintiles within each cohort. Quintile 1 corresponded to the most affluent neighborhoods, whereas quintile 5 referred to the most deprived neighborhoods.

Individual-level SES was determined based on DE for Medicare and Medicaid. Among Medicare beneficiaries, some patients with limited income and resources may be jointly enrolled in Medicaid if they meet eligibility criteria determined by their state of residence.^36^ Medicaid may help them cover costs and services not available through Medicare, such as long-term nursing facility services and home health services.^38^ Dual eligibility has been consistently used as an indicator for low income status.^37,38^ In this study, patients were considered dually eligible if they had at least 1 month of DE in the year prior to diagnosis.

### Outcomes

The primary outcome was overall survival, defined as time from cancer diagnosis to death from any cause or the end of the study (December 31, 2017), whichever came first. The secondary outcome was cancer-specific survival. Given that cause of death was not available for the year 2017, we set December 31, 2016, as the end of follow-up for cancer-specific survival analyses and censored patients at death if they died from other diseases rather than primary cancer.

### Patient Characteristics

The characteristics of patients included age at diagnosis (67-69, 70-74, 75-79, 80-84, or 85-94 years), sex (male or female), race and ethnicity (Black, Hispanic White, non-Hispanic White, or other [including Asian, Native American, races not identified as previous groups, and unknown]), marital status (yes, no, or unknown), cancer stage (I, II, III), Elixhauser comorbidity index (0, 1-2, or ≥3), surgery (yes or no), chemotherapy (yes or no), and radiotherapy (yes or no). Additionally, hormone receptor status (estrogen and progesterone receptors) was available for breast cancer and categorized as (1) either estrogen receptor or progesterone receptor was positive, (2) both estrogen receptor and progesterone receptor were negative, and (3) unknown. For prostate cancer, androgen deprivation therapy (yes, no) was also available. Race and ethnicity were commonly collected and identified by each cancer registry in the database. In this cohort, the overwhelming majority of Black patients were non-Hispanic; thus, non-Hispanic and Hispanic Black patients were combined as Black.

### Statistical Analysis

Patients’ characteristics were compared by ADI quintiles using the χ^2^ test for categorical variables and the Kruskal-Wallis test for continuous variables. Among each cancer type, differences of overall and cancer-specific survival between quintiles were assessed via the Kaplan-Meier method and the log-rank test.^49,50^ Only Q1, Q3, and Q5 are presented in Table 1 owing to space limitations.

**Table 1.** Demographic and Clinical Characteristics of Cancer Patients by Area Deprivation Index Quintiles

Characteristic^a^	No. (%)			*P* value^b^
	Q1	Q3	Q5	
**Breast cancer**				
No.	5375	5279	5206	
Age groups, y				
67-69	1006 (18.7)	922 (17.5)	851 (16.4)	<.001
70-74	1445 (26.9)	1456 (27.6)	1470 (28.2)	
75-79	1242 (23.1)	1205 (22.8)	1261 (24.2)	
80-84	926 (17.2)	946 (17.9)	911 (17.5)	
85-94	756 (14.1)	750 (14.2)	713 (13.7)	
Sex				
Male	NA	NA	NA	NA
Female	5375 (100.0)	5279 (100.0)	5206 (100.0)	
Race and ethnicity				
Black	120 (2.2)	312 (5.9)	734 (14.1)	<.001
Hispanic White	225 (4.2)	241 (4.6)	224 (4.3)	
Non-Hispanic White	4475 (83.3)	4496 (85.2)	4168 (80.1)	
Other^c^	555 (10.3)	230 (4.4)	80 (1.5)	
Marital status				
Yes	2710 (50.4)	2317 (43.9)	1975 (37.9)	<.001
No	2540 (47.3)	2689 (50.9)	2910 (55.9)	
Unknown	125 (2.3)	273 (5.2)	321 (6.2)	
Cancer stage				
I	3239 (60.3)	2992 (56.7)	2800 (53.8)	<.001
II	1656 (30.8)	1753 (33.2)	1823 (35.0)	
III	480 (8.9)	534 (10.1)	583 (11.2)	
Elixhauser comorbidity index				
0	2457 (45.7)	2086 (39.5)	1856 (35.7)	<.001
1-2	2027 (37.7)	2123 (40.2)	2158 (41.5)	
≥3	891 (16.6)	1070 (20.3)	1192 (22.9)	
Hormone receptor status				
ER+ or PR+	4663 (86.8)	4397 (83.3)	4226 (81.2)	<.001
ER− and PR−	589 (11.0)	661 (12.5)	707 (13.6)	
Unknown	123 (2.3)	221 (4.2)	273 (5.2)	
Surgery	5095 (94.8)	4964 (94.0)	4917 (94.5)	.20
Chemotherapy	1074 (20.0)	1158 (21.9)	1117 (21.5)	.11
Radiotherapy	3312 (61.6)	3003 (56.9)	2431 (46.7)	<.001
Dual eligibility for Medicare and Medicaid	499 (9.3)	627 (11.9)	1059 (20.3)	<.001
Follow-up time, median (IQR), y	7.3 (6.2-8.6)	7.1 (5.7-8.4)	6.8 (4.6-8.3)	<.001
Death	1594 (29.7)	1946 (36.9)	2240 (43.0)	<.001
Death from primary cancer	336 (6.3)	448 (8.5)	582 (11.2)	<.001
**Prostate cancer**				
No.	7209	7284	7109	
Age groups, y				
67-69	1779 (24.7)	1687 (23.2)	1674 (23.6)	.02
70-74	2619 (36.3)	2735 (37.6)	2529 (35.6)	
75-79	1686 (23.4)	1759 (24.2)	1682 (23.7)	
80-84	810 (11.2)	789 (10.8)	861 (12.1)	
85-94	315 (4.4)	314 (4.3)	363 (5.1)	
Sex				
Male	7209 (100.0)	7284 (100.0)	7109 (100.0)	NA
Female	NA	NA	NA	
Race and ethnicity				
Black	177 (2.5)	531 (7.3)	1364 (19.2)	<.001
Hispanic White	338 (4.7)	442 (6.1)	435 (6.1)	
Non-Hispanic White	5510 (76.4)	5775 (79.3)	5070 (71.3)	
Other^c^	1184 (16.4)	536 (7.4)	240 (3.4)	
Marital status				
Yes	5267 (73.1)	4843 (66.5)	4260 (59.9)	<.001
No	1122 (15.6)	1290 (17.7)	1683 (23.7)	
Unknown	820 (11.4)	1151 (15.8)	1166 (16.4)	
Stage				
I	<11 (<0.2)^d^	14 (0.2)	23 (0.3)	<.001
II	6590 (91.4)	6800 (93.4)	6725 (94.6)	
III	>608 (>8.4)	470 (6.5)	361 (5.1)	
Elixhauser comorbidity index				
0	3554 (49.3)	3345 (45.9)	2982 (42.0)	<.001
1-2	2781 (38.6)	2828 (38.8)	2779 (39.1)	
≥3	874 (12.1)	1111 (15.3)	1348 (19.0)	
Surgery	1909 (26.5)	1354 (18.6)	1034 (14.5)	<.001
Chemotherapy	2065 (28.6)	2464 (33.8)	2762 (38.9)	<.001
Radiotherapy	3263 (45.3)	3553 (48.8)	3322 (46.7)	<.001
ADT	1959 (27.2)	2362 (32.4)	2683 (37.7)	<.001
Dual eligibility for Medicare and Medicaid	469 (6.5)	554 (7.6)	989 (13.9)	<.001
Follow-up time, median (IQR), y	7.5 (6.5-8.8)	7.4 (6.3-8.8)	7.1 (5.7-8.5)	<.001
Death	1583 (22.0)	2134 (29.3)	2698 (38.0)	<.001
Death from primary cancer	198 (2.8)	268 (3.7)	336 (4.7)	<.001
**Lung cancer**				
No.	3274	3320	3311	
Age groups, y				
67-69	394 (12.0)	481 (14.5)	584 (17.6)	<.001
70-74	777 (23.7)	838 (25.2)	963 (29.1)	
75-79	830 (25.4)	867 (26.1)	839 (25.3)	
80-84	719 (22.0)	698 (21.0)	604 (18.2)	
85-94	554 (16.9)	436 (13.1)	321 (9.7)	
Sex				
Male	1560 (47.6)	1645 (49.5)	1713 (51.7)	.005
Female	1714 (52.4)	1675 (50.5)	1598 (48.3)	
Race and ethnicity				
Black	85 (2.6)	156 (4.7)	518 (15.6)	<.001
Hispanic White	131 (4)	184 (5.5)	99 (3.0)	
Non-Hispanic White	2605 (79.6)	2879 (86.7)	2661 (80.4)	
Other^c^	453 (13.8)	101 (3.0)	33 (1.0)	
Marital status				
Yes	1806 (55.2)	1653 (49.8)	1521 (45.9)	<.001
No	1401 (42.8)	1527 (46.0)	1642 (49.6)	
Unknown	67 (2.1)	140 (4.2)	148 (4.5)	
Stage				
I	1527 (46.6)	1469 (44.3)	1395 (42.1)	.03
II	271 (8.3)	287 (8.6)	294 (8.9)	
III	1476 (45.1)	1564 (47.1)	1622 (49.0)	
Elixhauser comorbidity index				
0	933 (28.5)	903 (27.2)	833 (25.2)	<.001
1-2	1407 (43.0)	1356 (40.8)	1349 (40.7)	
≥3	934 (28.5)	1061 (32.0)	1129 (34.1)	
Surgery	1440 (44.0)	1265 (38.1)	986 (29.8)	<.001
Chemotherapy	1052 (32.1)	1179 (35.5)	1122 (33.9)	.01
Radiotherapy	1014 (31.0)	1237 (37.3)	1306 (39.4)	<.001
Dual eligibility for Medicare and Medicaid	516 (15.8)	465 (14.0)	878 (26.5)	<.001
Follow-up time, median (IQR), y	2.1 (0.6-6.3)	1.7 (0.6-5.2)	1.3 (0.4-3.8)	<.001
Death	2576 (78.7)	2767 (83.3)	2934 (88.6)	<.001
Death from primary cancer	1825 (55.7)	1924 (58.0)	2057 (62.1)	<.001
**Colorectal cancer**				
No.	3935	3990	3826	
Age groups, y				
67-69	409 (10.4)	467 (11.7)	494 (12.9)	<.001
70-74	817 (20.8)	904 (22.7)	940 (24.6)	
75-79	872 (22.2)	899 (22.5)	898 (23.5)	
80-84	895 (22.7)	867 (21.7)	802 (21.0)	
85-94	942 (23.9)	853 (21.4)	692 (18.1)	
Sex				
Male	1788 (45.4)	1819 (45.6)	1715 (44.8)	.97
Female	2147 (54.6)	2171 (54.4)	2111 (55.2)	
Race and ethnicity				
Black	65 (1.7)	259 (6.5)	548 (14.3)	<.001
Hispanic White	225 (5.7)	210 (5.3)	176 (4.6)	
Non-Hispanic White	3046 (77.4)	3333 (83.5)	3033 (79.3)	
Other^c^	599 (15.2)	188 (4.7)	69 (1.8)	
Marital status				
Yes	2056 (52.2)	1916 (48.0)	1710 (44.7)	<.001
No	1789 (45.5)	1831 (45.9)	1876 (49.0)	
Unknown	90 (2.3)	243 (6.1)	240 (6.3)	
Stage				
I	1246 (31.7)	1292 (32.4)	1217 (31.8)	.66
II	1472 (37.4)	1436 (36.0)	1369 (35.8)	
III	1217 (30.9)	1262 (31.6)	1240 (32.4)	
Elixhauser comorbidity index				
0	1489 (37.8)	1421 (35.6)	1289 (33.7)	<.001
1-2	1501 (38.1)	1544 (38.7)	1437 (37.6)	
≥3	945 (24.0)	1025 (25.7)	1100 (28.7)	
Surgery	3588 (91.2)	3613 (90.6)	3419 (89.4)	.06
Chemotherapy	855 (21.7)	917 (23.0)	869 (22.7)	.44
Radiotherapy	417 (10.6)	428 (10.7)	413 (10.8)	.93
Dual eligibility for Medicare and Medicaid	659 (16.8)	586 (14.7)	850 (22.2)	<.001
Follow-up time, median (IQR), y	6.3 (2.7-8.1)	6.1 (2.2-7.8)	5.6 (1.8-7.7)	<.001
Death	2143 (54.5)	2394 (60.0)	2359 (61.7)	<.001
Death from primary cancer	763 (19.4)	917 (23.0)	926 (24.2)	<.001

Abbreviations: ADT, androgen deprivation therapy; ER, estrogen receptor; NA, not applicable; PR, progesterone receptor; Q1, quintile 1; Q3, quintile 3; Q5, quintile 5.

^a^ Continuous variables were presented as median (IQR) and categorical variables as No. (%). Percentages may not add up to 100% because of rounding. Only Q1, Q3, and Q5 are presented owing to space limitations.

^b^ *P* values were calculated using the Kruskal-Wallis test for continuous variables and the χ^2^ test for categorical variables.

^c^ Other consisted of Asian, Native American, other races not identified within these groups, and unknown race.

^d^ As per Surveillance, Epidemiology, and End Results–Medicare policy, numbers less than 11 must be suppressed to eliminate the potential for reidentification of persons with cancer.

We applied Cox proportional hazards regression to estimate hazard ratios (HRs) for the associations of ADI and DE with overall mortality and cancer-specific mortality.^51^ Regarding biological plausibility and change-in-estimate criterion (10% cutoff),^52,53^ the following were included as potential covariates: age, sex, marital status, cancer stage, Elixhauser comorbidity index, surgery, radiotherapy, and chemotherapy. Furthermore, hormone receptor status was considered for breast cancer, and androgen deprivation therapy was included for prostate cancer. Missing data for categorical variables were replaced with the level of “unknown,” whereas no missing data were observed for continuous variables. To calculate adjusted HRs, ADI and DE were both included in the models in addition to potential covariates. For sensitivity analysis, we also calculated adjusted HRs of ADI and DE without mutual adjustment. We used the Schoenfeld residuals method to test proportional hazards assumption and did not detect violations.^54^

To explore potential interactions between ADI and DE, we considered ADI as an ordinal variable, and the level of ADI quintiles (1 through 5) corresponded to the equivalent integer value (1 through 5). By DE status (yes vs no), we further estimated adjusted HRs of ADI by the increase of 1 quintile among 2 subgroups (DE vs non-DE beneficiaries) and assessed the interaction term using likelihood-ratio tests.^55^ In addition, we calculated distribution of DE by ADI and included this 2-way tabulation as eTable 1 in the Supplement.

We conducted all statistical analyses using SAS statistical software, version 9.4 (SAS Institute Inc) and R, version 4.0.2 (R Foundation for Statistical Computing) from January 23 to April 15, 2021. All *P* values were 2-sided, and *P* < .05 was considered as statistically significant. Point estimates were presented with 95% CIs. Multiple comparisons were not adjusted.

## Results

A total of 96 978 patients were analyzed. Of 25 968 female patients with breast cancer, median age at diagnosis was 76 years (IQR, 71-81 years); 1603 (6.2%) were Black, 21 725 (83.7%) were non-Hispanic White, and 2640 (10.2%) were of other race and ethnicity, including Hispanic; and 9545 (36.8%) died (median follow-up, 7.1 years [IQR, 5.5-8.4 years]). Of 35 150 male patients with prostate cancer, median age at diagnosis was 73 years (IQR, 70-77 years); 2884 (8.2%) were Black, 27 001 (76.8%) were non-Hispanic White, and 5265 (15.0%) were of other race and ethnicity, including Hispanic; and 10 431 (29.7%) died (median follow-up, 7.4 years [IQR, 6.3-8.7 years]). Of 16 684 patients with lung cancer, median age at diagnosis was 76 years (IQR, 71-81 years); 8412 (50.4%) were women; 1162 (7.0%) were Black, 13 926 (83.5%) were White, and 1596 (9.6%) were of other race and ethnicity, including Hispanic; and 13 984 (83.8%) died (median follow-up, 1.6 years [IQR, 0.5-5.1 years]). Of 19 176 patients with colorectal cancer, median age at diagnosis was 78 years (IQR, 72-84 years); 10 486 (54.7%) were women; 1233 (6.4%) were Black, 15 557 (81.1%) were White, and 2386 (12.4%) were of other race and ethnicity, including Hispanic; and 11 378 (59.3%) died (median follow-up, 6.1 years [IQR, 2.2-7.8 years]).

Regardless of cancer types, compared with those in the most affluent neighborhoods, patients living in the most disadvantaged neighborhoods were more likely to be Black, to be unmarried, to be DE beneficiaries, to have more comorbidities, and to die from any cause or primary cancer by the end of follow-up (Table 1). Patients with breast, lung, and colorectal cancer in the most disadvantaged neighborhoods tended to be younger and diagnosed at stage III (although stage was not significant for colorectal cancer). In contrast, among those with prostate cancer, those patients in the most disadvantaged neighborhoods were more likely to be older and have an early cancer stage. No clear patterns of differences in utilizing surgery, chemotherapy, and radiotherapy were observed among these cancer types across neighborhoods (Table 1).

**Figure 1.**
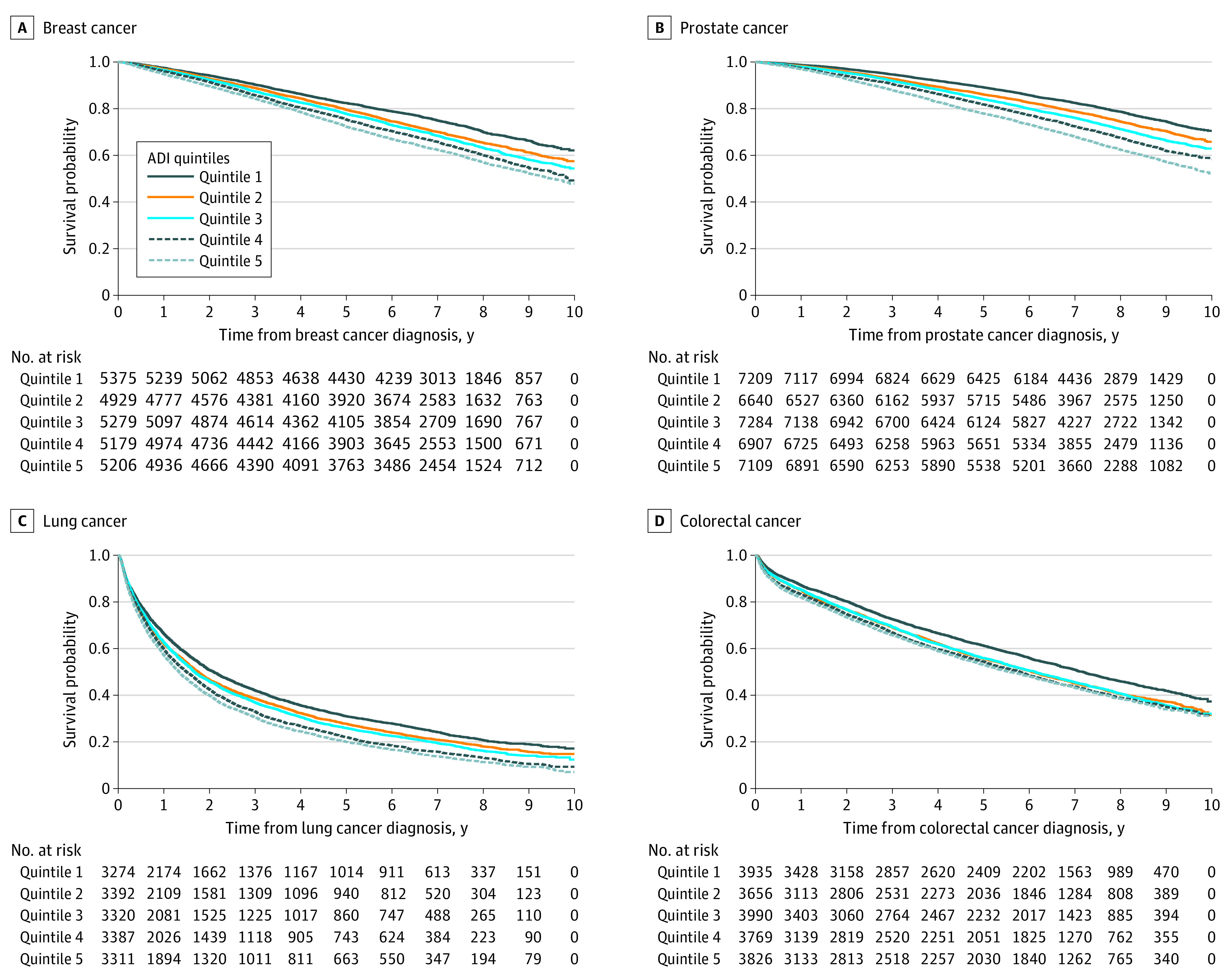
Kaplan-Meier Estimates for Overall Survival by Quintiles of Area Deprivation Index for Patients With Breast, Prostate, Lung, and Colorectal Cancer Overall survival by quintiles of ADI for breast (n = 25 968), prostate (n = 35 150), lung (n = 16 684), and colorectal cancer (n = 19 176). *P* values for each cancer were calculated using the log-rank test, and all were significant (*P* < .001). ADI indicates Area Deprivation Index.

Compared with those in the most affluent neighborhoods, patients living in the most disadvantaged neighborhoods had worse overall survival (Figure 1). For 5-year overall survival (Table 2), the estimate difference ranged from 8.1% (colorectal cancer) to 11.2% (prostate cancer). We observed a similar pattern for cancer-specific survival (Table 2; eFigure 2 in the Supplement), but the magnitudes of survival differences were noticeably different across cancer types. For example, the absolute percentage point difference in cancer-specific survival between quintile 1 and quintile 5 was small for prostate cancer (2.0%) but quite large for lung cancer (10.1%).

**Table 2.** Five-Year Overall and Cancer-Specific Survival by Area Deprivation Index Quintiles

ADI quintile	Overall survival, % (95% CI)				Cancer-specific survival, % (95% CI)			
	Breast	Prostate	Lung	CRC	Breast	Prostate	Lung	CRC
Q1	82.4 (81.4-83.4)	89.1 (88.4-89.8)	31.0 (29.4-32.6)	61.2 (59.7-62.7)	94.7 (94.0-95.2)	98.1 (97.8-98.4)	41.8 (40.0-43.6)	80.7 (79.4-82.0)
Q2	79.5 (78.4-80.6)	86.1 (85.2-86.9)	27.7 (26.2-29.2)	55.7 (54.1-57.3)	93.0 (92.3-93.7)	97.7 (97.3-98.0)	40.1 (38.3-41.9)	77.3 (75.8-78.7)
Q3	77.8 (76.6-78.9)	84.1 (83.2-84.9)	25.9 (24.4-27.4)	55.9 (54.4-57.5)	92.6 (91.9-93.3)	97.5 (97.1-97.9)	37.9 (36.1-39.7)	76.5 (75.0-77.8)
Q4	75.4 (74.2-76.5)	81.8 (80.9-82.7)	21.9 (20.6-23.3)	54.4 (52.8-56.0)	91.7 (90.9-92.5)	96.9 (96.4-97.3)	34.5 (32.7-36.3)	76.3 (74.8-77.7)
Q5	72.3 (71.0-73.5)	77.9 (76.9-78.8)	20.0 (18.7-21.4)	53.1 (51.5-54.6)	89.9 (89.1-90.8)	96.1 (95.6-96.5)	31.7 (30.0-33.5)	75.0 (73.5-76.4)

Abbreviations: ADI, Area Deprivation Index; CRC, colorectal cancer.

Among all cancer types, living in the most disadvantaged neighborhoods was significantly associated with unadjusted higher risk of overall mortality compared with living in the most affluent neighborhoods (eTable 2 in the Supplement). After comprehensive multivariable adjustment (Table 3), the associations were attenuated but remained statistically significant (adjusted HRs: 1.34 [95% CI, 1.26-1.43] for breast, 1.51 [95% CI, 1.42-1.62] for prostate, 1.21 [95% CI, 1.14-1.28] for lung, and 1.24 [95% CI, 1.17-1.32] for colorectal cancer). We observed a similar pattern for DE status, which was significantly associated with higher risk of overall mortality (adjusted HRs: 1.22 [95% CI, 1.15-1.29] for breast, 1.29 [95% CI, 1.21-1.38] for prostate, 1.14 [95% CI, 1.09-1.20] for lung, and 1.23 [95% CI, 1.17-1.29] for colorectal cancer).

**Table 3.** Adjusted Hazard Ratios Between Area Deprivation Index, Medicare-Medicaid Dual Eligibility, and Mortality^a^

	Breast^b^	*P* value	Prostate^c^	*P* value	Lung	*P* value	CRC	*P* value
**Overall mortality**								
ADI quintile								
Q1	1 [Reference]	NA	1 [Reference]	NA	1 [Reference]	NA	1 [Reference]	NA
Q2	1.12 (1.04-1.20)	.002	1.11 (1.04-1.19)	.002	1.08 (1.03-1.14)	.003	1.16 (1.09-1.23)	<.001
Q3	1.19 (1.12-1.27)	<.001	1.28 (1.19-1.36)	<.001	1.10 (1.04-1.16)	.001	1.18 (1.12-1.26)	<.001
Q4	1.31 (1.23-1.40)	<.001	1.36 (1.27-1.45)	<.001	1.21 (1.15-1.28)	<.001	1.23 (1.16-1.31)	<.001
Q5	1.34 (1.26-1.43)	<.001	1.51 (1.42-1.62)	<.001	1.21 (1.14-1.28)	<.001	1.24 (1.17-1.32)	<.001
DE								
No	1 [Reference]	NA	1 [Reference]	NA	1 [Reference]	NA	1 [Reference]	NA
Yes	1.22 (1.15-1.29)	<.001	1.29 (1.21-1.38)	<.001	1.14 (1.09-1.20)	<.001	1.23 (1.17-1.29)	<.001
**Cancer-specific mortality**								
ADI quintile								
Q1	1 [Reference]	NA	1 [Reference]	NA	1 [Reference]	NA	1 [Reference]	NA
Q2	1.20 (1.04-1.39)	.01	1.00 (0.82-1.22)	.98	1.05 (0.98-1.12)	.14	1.21 (1.09-1.33)	<.001
Q3	1.21 (1.05-1.40)	.008	1.22 (1.02-1.47)	.03	1.07 (1.00-1.14)	.04	1.27 (1.15-1.40)	<.001
Q4	1.31 (1.23-1.40)	<.001	1.36 (1.27-1.45)	<.001	1.21 (1.15-1.28)	<.001	1.23 (1.16-1.31)	<.001
Q5	1.50 (1.30-1.72)	<.001	1.38 (1.15-1.66)	<.001	1.16 (1.09-1.24)	<.001	1.33 (1.21-1.47)	<.001
DE								
No	1 [Reference]	NA	1 [Reference]	NA	1 [Reference]	NA	1 [Reference]	NA
Yes	1.23 (1.10-1.37)	<.001	1.29 (1.07-1.55)	.007	1.14 (1.08-1.21)	<.001	1.29 (1.19-1.40)	<.001

Abbreviations: ADI, Area Deprivation Index; CRC, colorectal cancer; DE, Medicare-Medicaid dual eligibility; NA, not applicable; Q1, quintile 1; Q2, quintile 2; Q3, quintile 3; Q4, quintile 4; Q5, quintile 5.

^a^ Adjusted for age, sex, race and ethnicity, marital status, stage, Elixhauser comorbidity index, surgery, radiotherapy, and chemotherapy. Area Deprivation Index and DE were mutually adjusted.

^b^ For breast cancer, hormone receptor status was additionally adjusted.

^c^ For prostate cancer, androgen deprivation therapy was additionally adjusted.

For cancer-specific survival, we detected a similar pattern. Across the 4 cancer types, living in the most disadvantaged neighborhoods and being a DE beneficiary were significantly associated with higher risk of dying from primary cancer (Table 2; eTable 2 in the Supplement).

**Figure 2.**
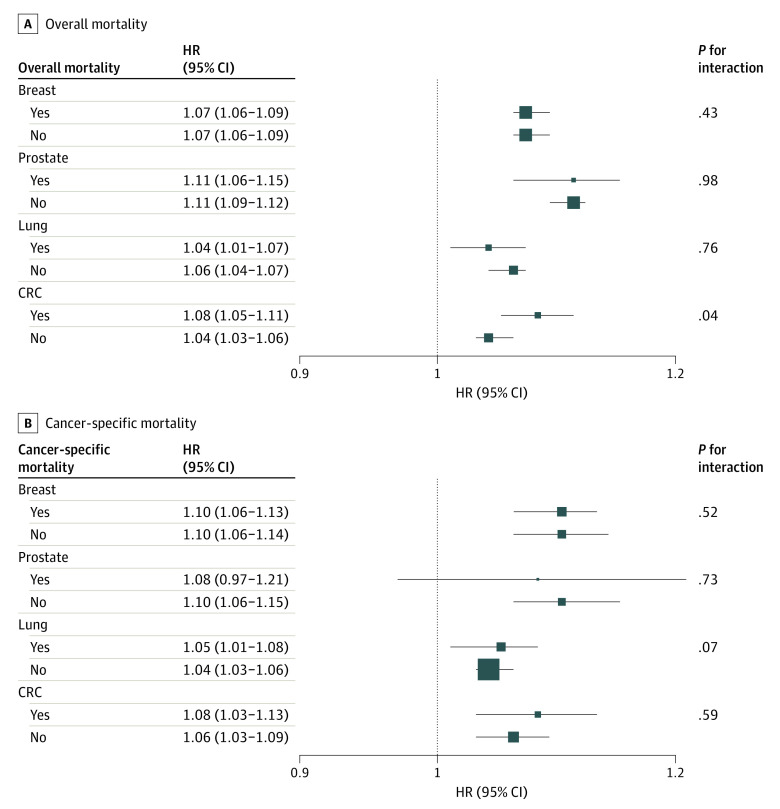
Associations Between Area Deprivation and Mortality According to Dual Eligibility Status (Yes vs No) by Cancer Types Adjusted for age, sex, race and ethnicity, marital status, cancer stage, Elixhauser comorbidity index, surgery, radiotherapy, and chemotherapy. Area Deprivation Index (ADI) and Medicare-Medicaid dual eligibility (DE) were mutually adjusted, and the interaction term ADI*DE was also included. For breast cancer, hormone receptor status was additionally adjusted. For prostate cancer, androgen deprivation therapy was additionally adjusted. *P* value for interaction was assessed using likelihood-ratio tests. ADI was evaluated as an ordinal variable, and each level (quintile) corresponded to the equivalent integer (from 1 through 5). CRC, colorectal cancer; HR, hazard ratio.

In sensitivity analyses, regardless of cancer types, simultaneously adjusting for both ADI and DE had little impact on the independent effect of each measure on survival (Table 3, eTable 3 in the Supplement). Additionally, of the 8 interactions of ADI and DE for overall and cancer-specific mortality among 4 cancer types (Figure 2), only 1 was significant, and the difference had limited clinical significance (HR, 1.08; 95% CI, 1.05-1.11 vs HR, 1.04; 95% CI, 1.03-1.06) among DE vs non-DE colorectal cancer patients, for adjusted HRs of ADI with overall mortality. Thus, these analyses may suggest that ADI and DE were independently associated with overall survival and cancer-specific survival.

## Discussion

In this study, we found that neighborhood and individual socioeconomic disadvantage were significantly and independently associated with worse overall and cancer-specific survival among patients with nonmetastatic breast, prostate, lung, and colorectal cancer.

Our study complements and expands on prior work in many important ways. First, instead of applying single-metric or one-off composite neighborhood SES features,^25,26,27,28,29,30,31,32^ we used the most recent version of ADI, a comprehensive composite measure to assess the complexity of neighborhood SES. Second, although individual SES information is frequently unavailable in large national databases,^17,56^ DE status among Medicare beneficiaries has been uniquely identified as a specific measure of patient-level low income,^36,37^ and we relied on it to reflect individual socioeconomic disadvantage. Third, using recent real-world big data, we examined multiple common cancers and assessed how neighborhood and individual socioeconomic disadvantage may together result in worse cancer outcomes, even after accounting for demographic, clinical, pathological, and treatment factors. The magnitudes of neighborhood and individual socioeconomically disadvantaged status are comparable to the effects of contemporary adjuvant therapies for these common cancers.^57,58,59,60^ Thus, along with investing in treatment development, these novel findings support the need for policies for ongoing investments in disadvantaged neighborhoods and low-income households.

This study is among the first to our knowledge to use national data, as most prior studies investigated the link between ADI and cancer using primarily regional databases. Neighborhood disadvantage has been reported to be associated with (1) increased cancer prevalence, incidence, and mortality^8,46^; (2) obesity and inferior overall survival among pediatric patients with acute lymphoblastic leukemia^61,62^; (3) lack of adjuvant therapy among patients with localized pancreatic cancer^63^; and (4) anxiety among patients with advanced cancer.^64^ One SEER study^8^ reported higher ADI (neighborhood disadvantage) to be associated with worse overall survival among cancer patients diagnosed between 1988 and 1999: HRs (highest decile vs lowest decile) were 1.68 (95% CI, 1.57-1.79) for breast, 1.57 (95% CI, 1.46-1.68) for prostate, 1.29 (95% CI, 1.23-1.36) for colorectal, and 1.56 (95% CI, 1.54-1.59) for all cancers. However, such survival estimates were not adjusted for cancer stage and treatment and may not accurately reflect the associations of neighborhood disadvantage with cancer survival, given that stage and treatment are key factors influencing the prognosis of cancer patients.^65^ From 55 clinical trials for 15 cancer types (1985-2012), 1 recent study^45^ identified 41 109 patients with cancer and combined them as a single cohort for the analysis. Compared with the lowest ADI quintile, patients with cancer living in the most deprived neighborhoods (highest quintile) had worse overall survival (HR, 1.28; 95% CI, 1.20-1.37), progression-free survival (HR, 1.20; 95% CI, 1.13-1.28), and cancer-specific survival (HR, 1.27; 95% CI, 1.18-1.37), suggesting that even in this group of trial participants who initially all had access to high-quality cancer care, there were still survival disparities across strata of neighborhood SES. Given that all cancer types were combined and separate analyses were not conducted, it was unclear which cancers were primarily subject to neighborhood socioeconomic disadvantage. In addition, only 8% of American adult cancer patients enrolled in clinical trials owing to multiple structural and clinical barriers for trial participants.^66,67^ Thus, the generalizability of such results has not been examined until now. Using a large national cancer database, our findings may be generalizable to a broader neighborhood and cancer population across the country. Our study adds to prior findings by demonstrating that (1) survival disparities persist after adjustment for demographic, clinical, pathological, treatment, and individual SES factors in real-world data; and (2) the survival of patients with the 4 most common cancers is subject to neighborhood disadvantage as measured by the ADI.

Furthermore, in comparing 5-year cancer-specific survival across the most affluent and deprived neighborhoods, we noticed a small difference for prostate cancer but a large one for lung cancer. In this study, at least 90% of prostate cancer cases were diagnosed at stage I and cancer-specific survival was at least 96% regardless of neighborhood SES, suggesting that patients were very likely to be cured from primary prostate cancer.^68^ However, patients with lung cancer living in the most deprived neighborhood were more likely to be diagnosed at stage III and less likely to get surgery, which was considered to be the most curative treatment.^69^ Thus, a large difference in cancer-specific survival between neighborhoods was observed among patients with lung cancer. Future studies in other cancer types are also needed to explore the differences of cancer-specific survival estimates between neighborhoods.

In large national cancer databases,^17,70^ individual-level SES information is frequently unavailable, whereas such information is available in the SEER-Medicare database. Although DE has been consistently considered as a reliable indicator for low income,^37,38^ only 4 prior studies have looked into DE status and cancer survival: 3 used state-level data (Ohio, Michigan, and North Carolina), and only 1 included all SEER states.^71,72,73,74^ In these studies, DE status was reported to be associated with inferior overall survival after diagnosis of prostate, lung, and gynecologic cancer^71,72,73,74^ and worse colorectal cancer–specific survival.^72^ Owing to limited prior work and lack of replicability, our study complements previous studies by evaluating the most common cancers through recent national SEER-Medicare data and reports the associations of individual socioeconomic disadvantage with inferior survival among patients with breast, prostate, lung, and colorectal cancer. Thus, in addition to adjusting for neighborhood-level SES, future studies using national cancer databases should also adjust for individual-level SES measures if they are available or could be obtained from external resources.

Cancer treatment confers a large financial burden on patients and the health system.^75,76^ Although patients’ medical expenses may be largely covered by Medicare and/or Medicaid, we still observed persistent survival disparities associated with neighborhood and individual SES disparities, even after accounting for cancer treatment. The recent COVID-19 pandemic has exacerbated long-standing economic and health inequalities^77,78^ and has exposed cancer patients, especially those from neighborhood and individual socioeconomically disadvantaged backgrounds, to greater risk of death.^79,80^ Health inequities in cancer survival related to neighborhood and individual SES status are symptoms of deeply rooted structural and systematic barriers in policy, education, health care, employment, insurance, and the justice system, as well as underlying racism and classism. For patients living in poverty and/or in deprived neighborhoods, these barriers can manifest in many ways that negatively influence cancer survival,^25,26,27,28,29,30,31,32^ including financial toxicity (ie, difficulty related to the cost of medical care) after cancer diagnosis,^81^ lack of access to physicians and health care resources,^20^ unequal treatment in health care,^82^ inefficacious referral systems,^21^ mistrust between providers and patients,^22^ poor social support network,^23^ and barriers to travel for initial and follow-up care.^24^ Our findings may support policies for ongoing investments in disadvantaged neighborhoods and low-income households, which could increase their opportunities for healthy and safe living and expand their access to timely cancer treatment.

### Limitations

This study has several limitations. Although ADI is a comprehensive composite measure to assess neighborhood socioeconomic disadvantage, it may not reflect every aspect of neighborhood SES. However, in line with other studies using alternative composite measures of neighborhood SES (such as the Yost index),^83^ inferior survival was observed among patients with breast, prostate, lung, and colorectal cancer.^84,85,86,87^ A similar limitation also applies to DE status. Low income is an important attribute of disadvantaged SES, but it may not manifest each facet within individual SES. However, our findings add to limited prior work and replicability in research. Additionally, we focused only on the 4 most commonly diagnosed cancers; thus, our findings may not be generalizable to other cancer types. Furthermore, there are some important limitations to the SEER-Medicare database. Our analysis was restricted to older Medicare beneficiaries with fee-for-service coverage, so our results may not be generalizable to younger patients or those with other types of insurance.

## Conclusions

The findings of this cohort study suggest that patients with breast, prostate, lung, and colorectal cancer experienced worse survival if coming from neighborhood or individual socioeconomically disadvantaged backgrounds. Such disparities persisted even after accounting for demographic, clinical, pathological, and treatment factors. Policies for ongoing investments in low-resource neighborhoods and low-income households are needed to improve cancer outcomes and reduce health disparities. Future studies should examine other common cancers to help health professionals and policy makers better understand the differences in cancer outcomes.

## Notes

Supplement. **eFigure 1.** Sample Construction for Patients Diagnosed With Breast (eFigure 1A), Prostate (eFigure 1B), Lung (eFigure 1C), and Colorectal Cancer (eFigure 1D) **eTable 1.** Distribution of Medicare-Medicaid Dual Eligibility by Area Deprivation Index Among Cancer Patients **eFigure 2.** Kaplan-Meier Estimates for Cancer-Specific Survival by Quintiles of Area Deprivation Index among Breast (1A), Prostate (1B), Lung (1C) and Colorectal (1D) Cancer **eTable 2.** Unadjusted Hazard Ratios of Area Deprivation Index and Medicare-Medicaid Dual Eligibility Associated with Mortality **eTable 3.** Non-Mutually Adjusted Hazard Ratios of Area Deprivation Index and Medicare-Medicaid Dual Eligibility Associated with Mortality
