# Supplementary material for: Neighborhood and Individual Socioeconomic Disadvantage and Survival Among Patients With Nonmetastatic Common Cancers
Source: JAMA Netw Open. 2021 Dec 17;4(12):e2139593. doi: 10.1001/jamanetworkopen.2021.39593 (PMC8683967; doi:10.1001/jamanetworkopen.2021.39593)
Supplement: Supplement. — eFigure 1. Sample Construction for Patients Diagnosed With Breast (eFigure 1A), Prostate (eFigure 1B), Lung (eFigure 1C), and Colorectal Cancer (eFigure 1D) eTable 1. Distribution of Medicare-Medicaid Dual Eligibility by Area Deprivation Index Among Cancer Patients eFigure 2. Kaplan-Meier Estimates for Cancer-Specific Survival by Quintiles of Area Deprivation Index among Breast (1A), Prostate (1B), Lung (1C) and Colorectal (1D) Cancer eTable 2. Unadjusted Hazard Ratios of Area Deprivation Index and Medicare-Medicaid Dual Eligibility Associated with Mortality eTable 3. Non-Mutually Adjusted Hazard Ratios of Area Deprivation Index and Medicare-Medicaid Dual Eligibility Associated with Mortality [file jamanetwopen-e2139593-s001.pdf]

Supplemental Online Content

Cheng E, Soulos PR, Irwin ML, et al. Neighborhood and individual socioeconomic disadvantage and survival among patients with nonmetastatic common cancers. *JAMA Netw Open.* 2021;4(12):e2139593. doi:10.1001/jamanetworkopen.2021.39593

eFigure 1. Sample Construction for Patients Diagnosed With Breast (eFigure 1A), Prostate (eFigure 1B), Lung (eFigure 1C), and Colorectal Cancer (eFigure 1D)

eTable 1. Distribution of Medicare-Medicaid Dual Eligibility by Area Deprivation Index Among Cancer Patients

eFigure 2. Kaplan-Meier Estimates for Cancer-Specific Survival by Quintiles of Area Deprivation Index among Breast (1A), Prostate (1B), Lung (1C) and Colorectal (1D) Cancer

eTable 2. Unadjusted Hazard Ratios of Area Deprivation Index and Medicare-Medicaid Dual Eligibility Associated with Mortality

eTable 3. Non-Mutually Adjusted Hazard Ratios of Area Deprivation Index and Medicare-Medicaid Dual Eligibility Associated with Mortality

This supplemental material has been provided by the authors to give readers additional information about their work.

© 2021 Cheng E et al. *JAMA Network Open.*

eFigure 1. Sample Construction for Patients Diagnosed With Breast (eFigure 1A), Prostate (eFigure 1B), Lung (eFigure 1C), and Colorectal Cancer (eFigure 1D)

A

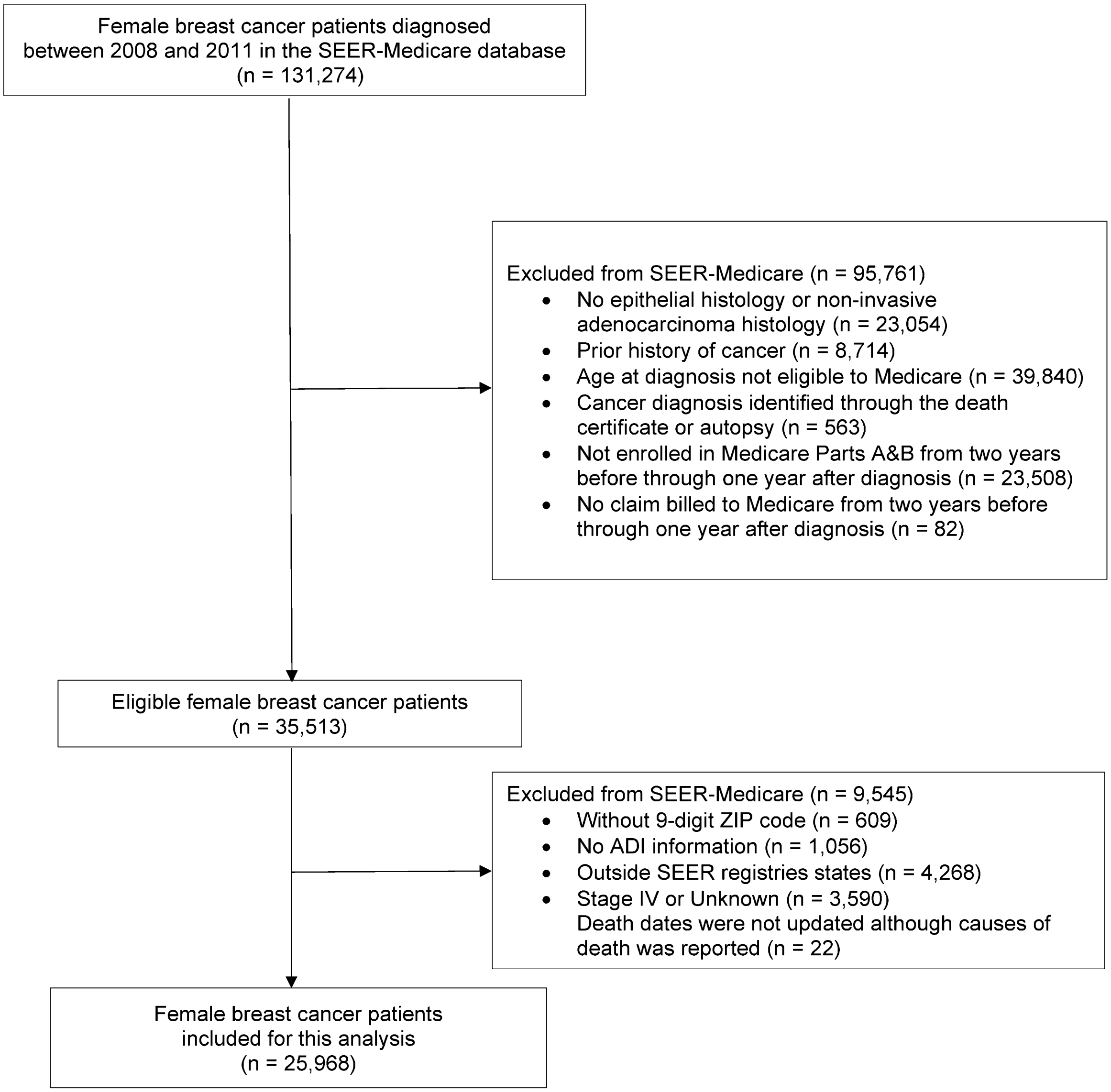

© 2021 Cheng E et al. *JAMA Network Open.*

B

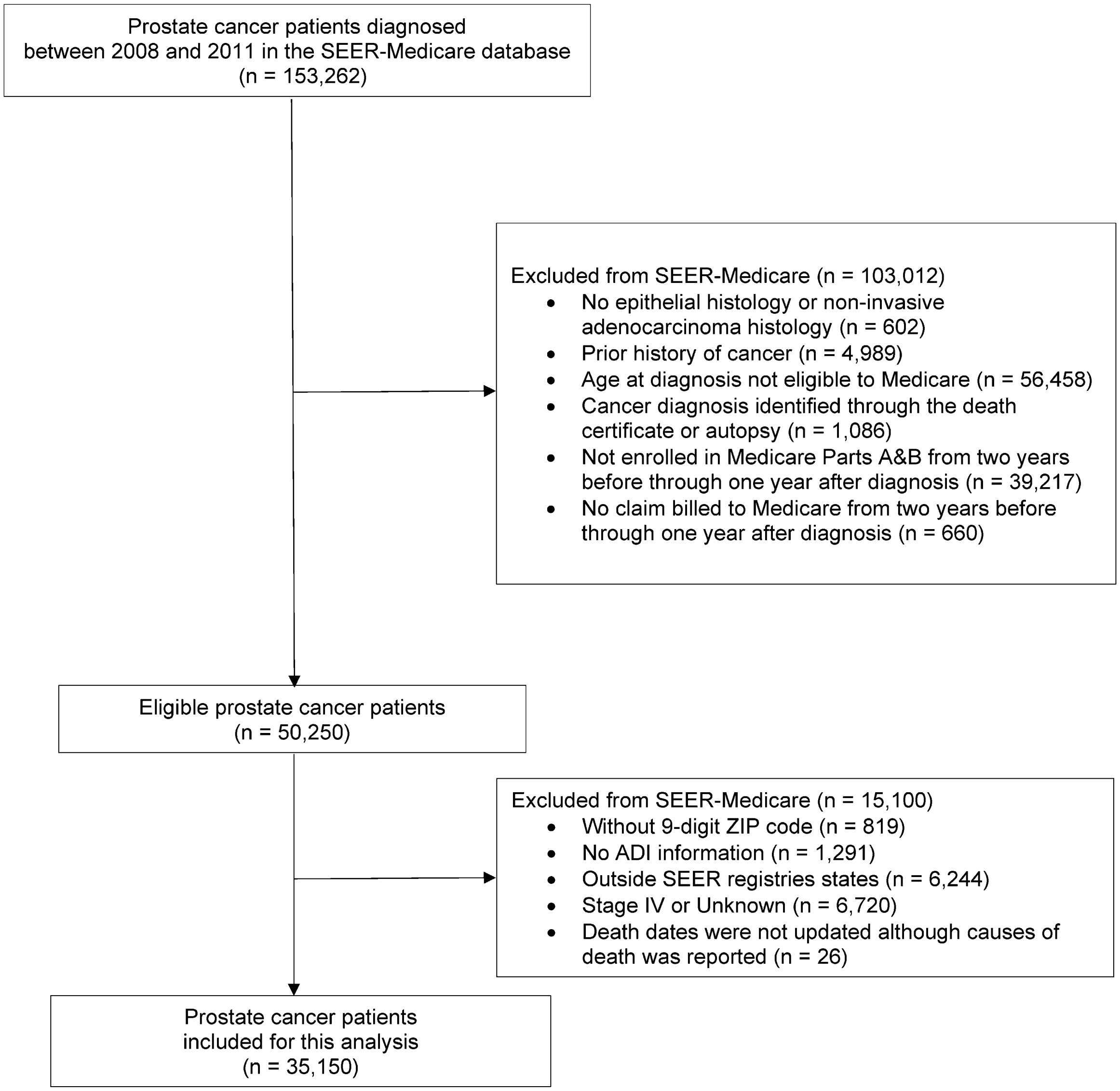

© 2021 Cheng E et al. *JAMA Network Open.*

C

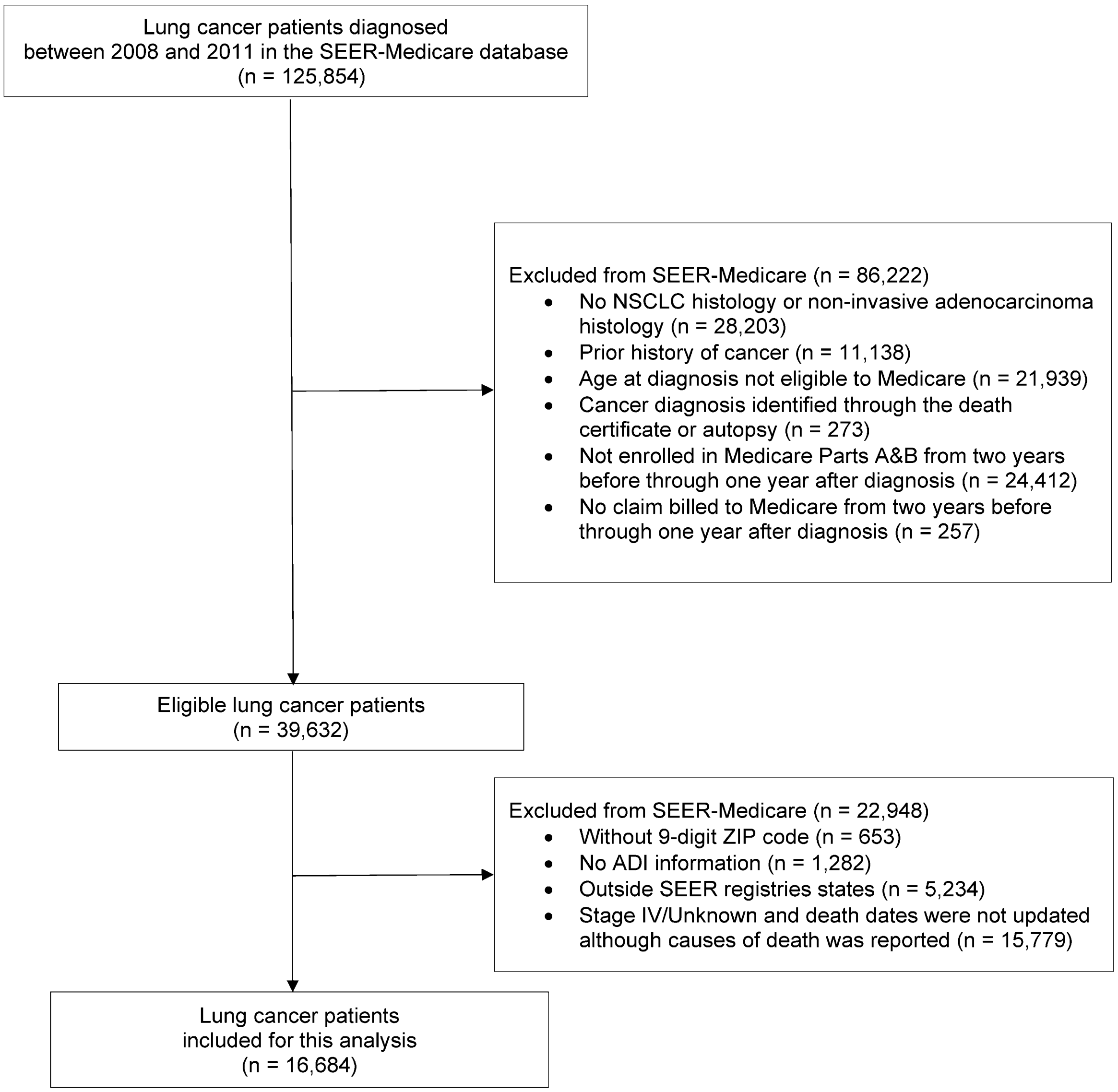

© 2021 Cheng E et al. *JAMA Network Open.*

D

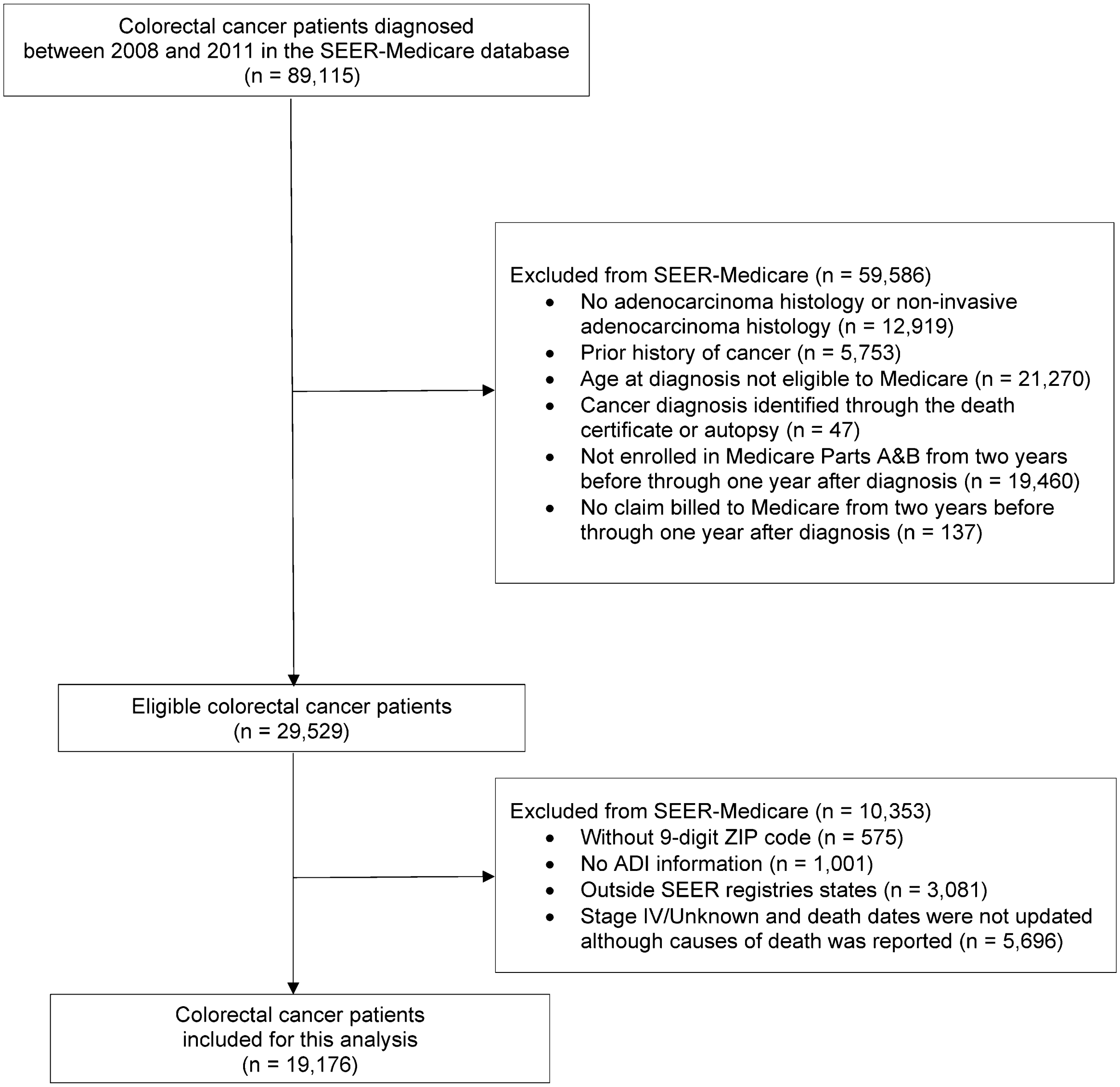

© 2021 Cheng E et al. *JAMA Network Open.*

eTable 1. Distribution of Medicare-Medicaid Dual Eligibility by Area Deprivation Index Among Cancer Patients

Breast			ADI			P-value [a]
Dual Eligibility	Q1	Q2	Q3	Q4	Q5	<0.001
No	4,876 (90.7)	4,252 (86.3)	4,652 (88.1)	4,525 (87.4)	4,147 (79.7)	
Yes	499 (9.3)	677 (13.7)	627 (11.9)	654 (12.6)	1,059 (20.3)	
Prostate						
Dual Eligibility	Q1	Q2	Q3	Q4	Q5	<0.001
No	6,740 (93.5)	5,996 (90.3)	6,730 (92.4)	6,384 (92.4)	6,120 (86.1)	
Yes	469 (6.5)	644 (9.7)	554 (7.6)	523 (7.6)	989 (13.9)	
Lung						
Dual Eligibility	Q1	Q2	Q3	Q4	Q5	<0.001
No	2,758 (84.2)	2,839 (83.7)	2,855 (86.0)	2,811 (83.0)	2,433 (73.5)	
Yes	516 (15.8)	553 (16.3)	465 (14.0)	576 (17.01)	878 (26.5)	
Colorectal						
Dual Eligibility	Q1	Q2	Q3	Q4	Q5	<0.001
No	3,276 (83.3)	2,964 (81.1)	3,404 (85.3)	3,172 (84.2)	2,976 (77.8)	
Yes	659 (16.7)	692 (18.9)	586 (14.7)	597 (15.8)	850 (22.2)	

Abbreviations: ADI, Area Deprivation Index; Q1, quintile 1; Q2, quintile 2; Q3, quintile 3; Q4, quintile 4; Q5, quintile 5

[a] P-values were calculated using the chi-squared test.

© 2021 Cheng E et al. *JAMA Network Open.*

eFigure 2. Kaplan-Meier Estimates for Cancer-Specific Survival by Quintiles of Area Deprivation Index Among Breast (1A), Prostate (1B), Lung (1C) and Colorectal (1D) Cancer

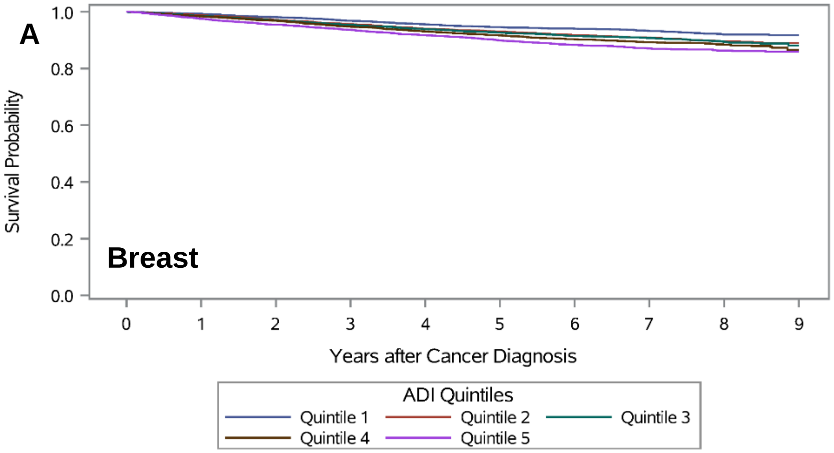

Quintile 1	5375	5239	5062	4853	4638	4430	3176	1979	901	0
Quintile 2	4929	4777	4576	4381	4160	3920	2753	1756	813	0
Quintile 3	5279	5097	4874	4614	4362	4105	2894	1820	835	0
Quintile 4	5179	4974	4736	4442	4166	3903	2733	1648	738	0
Quintile 5	5206	4936	4666	4390	4091	3763	2642	1656	779	0

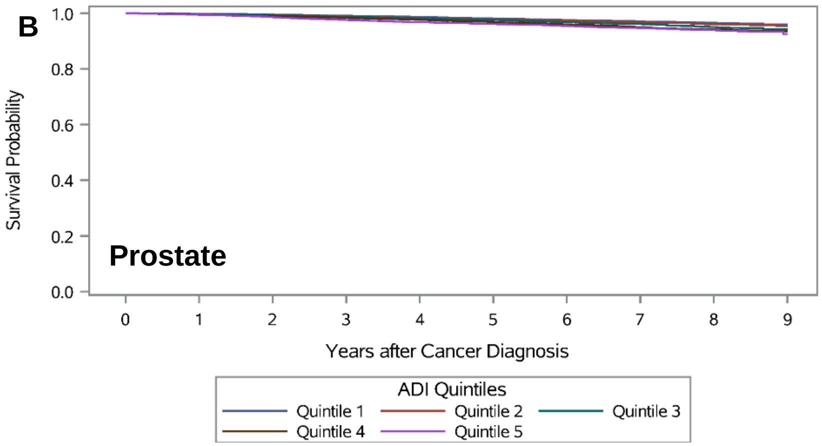

Quintile 1	7209	7117	6994	6824	6629	6425	4620	3016	1506	0
Quintile 2	6640	6527	6360	6162	5937	5715	4165	2718	1328	0
Quintile 3	7284	7138	6942	6700	6424	6124	4452	2908	1444	0
Quintile 4	6907	6725	6493	6258	5963	5651	4113	2658	1229	0
Quintile 5	7109	6891	6590	6253	5890	5538	3944	2497	1183	0

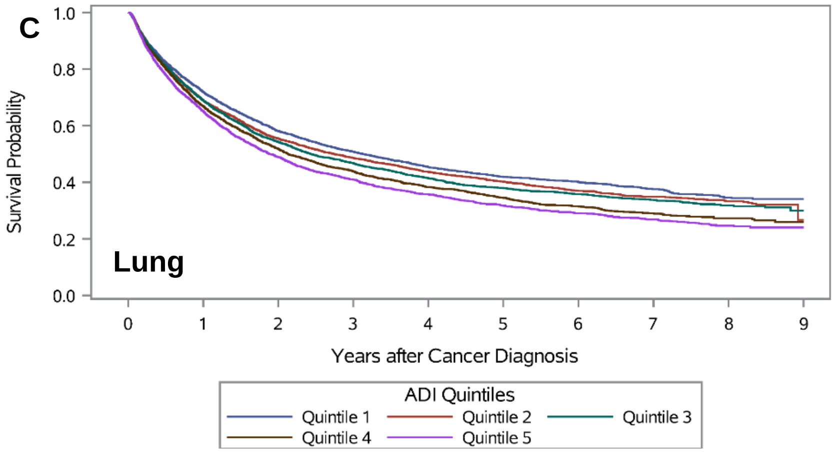

Quintile 1	3274	2174	1662	1376	1167	1014	714	397	165	0
Quintile 2	3392	2109	1581	1309	1096	940	592	347	150	0
Quintile 3	3320	2081	1525	1225	1017	860	569	321	128	0
Quintile 4	3387	2026	1439	1118	905	743	456	264	114	0
Quintile 5	3311	1894	1320	1011	811	663	411	234	94	0

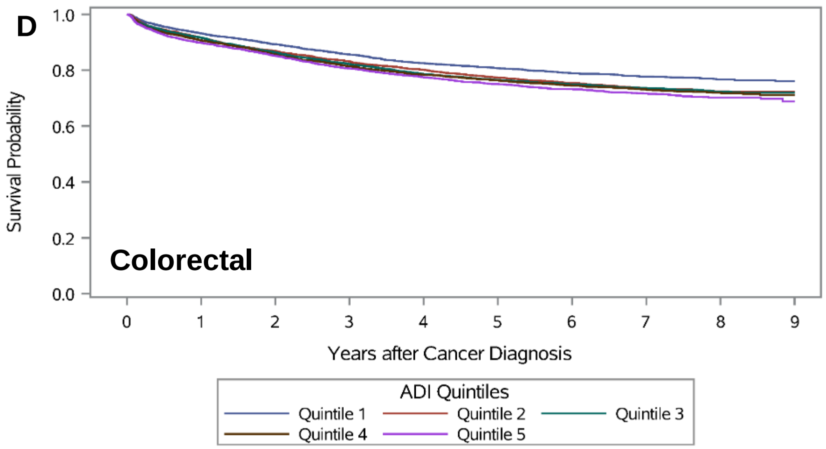

Quintile 1	3935	3428	3158	2857	2620	2409	1719	1096	524	0
Quintile 2	3656	3113	2806	2531	2273	2036	1434	900	423	0
Quintile 3	3990	3403	3060	2764	2467	2232	1582	992	450	0
Quintile 4	3769	3139	2819	2520	2251	2051	1411	861	392	0
Quintile 5	3826	3133	2813	2518	2257	2030	1413	854	388	0

Abbreviation: ADI, area deprivation index. Cancer-specific survival by quintiles of ADI for breast (Figure 1A; N = 25,968), prostate (Figure 1B; N = 35,150), lung (Figure 1C; N = 16,684), and colorectal cancer (Figure 1D; N = 19,176). *P*-values for each cancer were calculated using the log-rank test, and all were significant (*P*-value <0.001).

© 2021 Cheng E et al. *JAMA Network Open.*

eTable 2. Unadjusted Hazard Ratios of Area Deprivation Index and Medicare-Medicaid Dual Eligibility Associated with Mortality

	Overall Mortality										
	Breast	*P*-value		Prostate	*P*-value		Lung	*P*-value		CRC	*P*-value
ADI											
Q1	1.00			1.00			1.00			1.00	
Q2	1.20 (1.12-1.29)	<0.001		1.22 (1.14-1.30)	<0.001		1.11 (1.05-1.17)	<0.001		1.16 (1.09-1.23)	<0.001
Q3	1.30 (1.22-1.39)	<0.001		1.40 (1.31-1.49)	<0.001		1.15 (1.09-1.22)	<0.001		1.17 (1.10-1.24)	<0.001
Q4	1.46 (1.36-1.55)	<0.001		1.62 (1.52-1.73)	<0.001		1.29 (1.22-1.36)	<0.001		1.23 (1.16-1.30)	<0.001
Q5	1.61 (1.51-1.71)	<0.001		1.95 (1.83-2.07)	<0.001		1.38 (1.31-1.46)	<0.001		1.26 (1.19-1.33)	<0.001
DE											
No	1.00			1.00			1.00			1.00	
Yes	1.70 (1.61-1.79)	<0.001		1.55 (1.47-1.65)	<0.001		1.31 (1.26-1.37)	<0.001		1.34 (1.28-1.40)	<0.001
	Cancer-Specific Mortality										
	Breast[b]	*P*-value		Prostate[c]	*P*-value		Lung	*P*-value		CRC	*P*-value
ADI											
Q1	1.00			1.00			1.00			1.00	
Q2	1.36 (1.18-1.58)	<0.001		1.12 (0.92-1.37)	0.24		1.07 (1.01-1.14)	0.03		1.21 (1.09-1.33)	<0.001
Q3	1.40 (1.22-1.62)	<0.001		1.39 (1.15-1.67)	<0.001		1.11 (1.05-1.19)	<0.001		1.24 (1.12-1.36)	<0.001
Q4	1.60 (1.39-1.84)	<0.001		1.70 (1.42-2.04)	<0.001		1.23 (1.15-1.31)	<0.001		1.27 (1.15-1.40)	<0.001
Q5	1.93 (1.68-2.20)	<0.001		1.89 (1.59-2.25)	<0.001		1.32 (1.24-1.41)	<0.001		1.35 (1.23-1.49)	<0.001
DE											
No	1.00			1.00			1.00			1.00	
Yes	1.93 (1.75-2.14)	<0.001		1.52 (1.29-1.79)	<0.001		1.31 (1.24-1.37)	<0.001		1.38 (1.28-1.49)	<0.001

Abbreviations: ADI, area deprivation index; DE, Medicare-Medicaid dual eligibility; CRC, colorectal cancer; Q1, quintile 1; Q2, quintile 2; Q3, quintile 3; Q4, quintile 4; Q5, quintile 5.

© 2021 Cheng E et al. *JAMA Network Open.*

eTable 3. Non-Mutually Adjusted Hazard Ratios[a] of Area Deprivation Index and Medicare-Medicaid Dual Eligibility Associated with Mortality

	Overall Mortality										
	Breast[b]	*P*-value		Prostate[c]	*P*-value		Lung	*P*-value		CRC	*P*-value
ADI											
Q1	1.00			1.00			1.00			1.00	
Q2	1.12 (1.05-1.20)	<0.001		1.12 (1.05-1.20)	0.001		1.08 (1.03-1.14)	0.003		1.16 (1.09-1.23)	<0.001
Q3	1.19 (1.12-1.28)	<0.001		1.28 (1.19-1.36)	<0.001		1.10 (1.04-1.16)	0.001		1.18 (1.11-1.25)	<0.001
Q4	1.32 (1.23-1.41)	<0.001		1.36 (1.28-1.46)	<0.001		1.21 (1.15-1.28)	<0.001		1.23 (1.16-1.31)	<0.001
Q5	1.36 (1.27-1.46)	<0.001		1.53 (1.44-1.64)	<0.001		1.22 (1.16-1.29)	<0.001		1.25 (1.18-1.33)	<0.001
DE											
No	1.00			1.00			1.00			1.00	
Yes	1.24 (1.17-1.31)	<0.001		1.32 (1.24-1.41)	<0.001		1.16 (1.10-1.21)	<0.001		1.24 (1.17-1.30)	<0.001
	Cancer-Specific Mortality										
	Breast[b]	*P*-value		Prostate[c]	*P*-value		Lung	*P*-value		CRC	*P*-value
ADI											
Q1	1.00			1.00			1.00			1.00	
Q2	1.21 (1.05-1.40)	0.009		1.01 (0.83-1.23)	0.94		1.05 (0.98-1.12)	0.15		1.21 (1.10-1.34)	<0.001
Q3	1.22 (1.05-1.40)	0.007		1.22 (1.02-1.47)	0.03		1.07 (1.00-1.14)	0.04		1.27 (1.15-1.40)	<0.001
Q4	1.35 (1.17-1.55)	<0.001		1.35 (1.12-1.62)	0.001		1.17 (1.10-1.25)	<0.001		1.28 (1.16-1.41)	<0.001
Q5	1.52 (1.33-1.75)	<0.001		1.40 (1.17-1.68)	<0.001		1.17 (1.10-1.25)	<0.001		1.35 (1.22-1.49)	<0.001
DE											
No	1.00			1.00			1.00			1.00	
Yes	1.26 (1.12-1.41)	<0.001		1.30 (1.08-1.57)	0.005		1.15 (1.09-1.22)	<0.001		1.30 (1.20-1.41)	<0.001

Abbreviations: ADI, area deprivation index; DE, Medicare-Medicaid dual eligibility; CRC, colorectal cancer; Q1, quintile 1; Q2, quintile 2; Q3, quintile 3; Q4, quintile 4; Q5, quintile 5.

[a] Adjustment for age, sex, race/ethnicity, marital status, stage. Elixhauser comorbidity index, surgery, radiotherapy, and chemotherapy. ADI and dual eligibility were not mutually adjusted.

[b] For breast cancer, hormone receptor status was additionally adjusted.

[c] For prostate cancer, androgen deprivation therapy was additionally adjusted.

© 2021 Cheng E et al. *JAMA Network Open.*
